# Maternal anthropometry: trends and inequalities in four population-based birth cohorts in Pelotas, Brazil, 1982–2015

**DOI:** 10.1093/ije/dyy278

**Published:** 2019-03-18

**Authors:** Bernardo L Horta, Fernando C Barros, Natália P Lima, Maria C F Assunção, Iná S Santos, Marlos R Domingues, Cesar G Victora, Pelotas Cohorts Study Group, Aluisio J D Barros, Alicia Matijasevich, Ana M B Menezes, Andrea Dâmaso Bertoldi, Diego G Bassani, Fernando C Wehrmeister, Helen Gonçalves, Joseph Murray, Luciana Tovo-Rodrigues, Mariangela F Silveira, Pedro R C Hallal

**Affiliations:** 1 Federal University of Pelotas, Brazil; 2 University of São Paulo, Brazil; 3 University of Toronto, Canada; 1 Postgraduate Program in Epidemiology, Federal University of Pelotas; 2 Postgraduate Program in Health and Behavior, Catholic University of Pelotas, Pelotas, Brazil

**Keywords:** Nutritional status, anthropometry, maternal health, pregnancy, socioeconomic factors, cohort studies

## Abstract

**Background:**

Pre-pregnancy nutritional status and weight gain during pregnancy have short- and long-term consequences for the health of women and children. This study was aimed at evaluating maternal height,- and overweight or obesity at the beginning of the pregnancy and gestational weight gain, according to socioeconomic status and maternal skin colour of mothers in Pelotas, a southern Brazilian city, in 1982, 1993, 2004 and 2015.

**Methods:**

In 1982, 1993, 2004 and 2015, the maternity hospitals in Pelotas were visited daily, all deliveries were identified and mothers who lived in the urban area of the city were interviewed. Maternal weight at the beginning of the pregnancy was self-reported by the mother or obtained from the antenatal card. Maternal height was collected from the maternity records or measured by the research team. Overweight or obesity was defined by a body mass index ≥25 kg/m^2^. Gestational weight gain was evaluated according to the Institute of Medicine guidelines.

**Results:**

In the four cohorts, we evaluated 19 931 women. From 1982 to 2015, the prevalence of overweight or obesity at the beginning of the pregnancy increased from 22.1% to 47.0% and height increased by an average of 5.2 cm, whereas gestational weight gain did not change. Socioeconomic status was positively associated with maternal height, and the difference between the poorest and the wealthiest decreased. Overweight or obesity was lower among those mothers in the extreme categories of family income.

**Conclusions:**

Over the 33-year span, mothers were taller at the beginning of the pregnancy, but the prevalence of overweight or obesity more than doubled.

**Key Messages**

- Maternal height increased byan average of 5.2 cm from 1982 to 2015 and inequalities by socioeconomic status decreased.
- Increase in weight was greater than that observed for height, and prevalence of overweight or obesity at the beginning of the pregnancy increased from 22.1% to 47.0%
- Prevalence of maternal underweight at the beginning of the pregnancy decreased, but the reduction was higher among the wealthiest mothers and inequalities by socioeconomic status increased.

## Introduction

Maternal nutritional status during pregnancy is often evaluated through anthropometric indicators such as pre-pregnancy height, weight and body mass index (BMI), and by weight gain during pregnancy. Maternal height results from the interaction of the genetic potential for growth with early life conditions,^1^ whereas maternal pre-pregnancy weight and BMI reflect nutritional status before conception. Gestational weight gain, on the other hand, depends on health and nutrition during pregnancy.

Malnutrition before and during pregnancy may have short- and long-term consequences. Maternal height, weight, BMI and gestational weight gain are positively associated with intrauterine growth and birthweight.^2^ Maternal height is also associated with long-term consequences, being positively related to the offspring’s human capital^3^ and linear growth.^4^ Maternal underweight is a risk factor for several perinatal outcomes.^5^ On the other hand, pre-pregnancy overweight or obesity increases the risk of stillbirth and infant mortality,^6^ preterm birth and large-for-gestational- age babies.^7^ Excessive weight gain during pregnancy is a risk factor for adverse fetal and maternal outcomes.^8^ Furthermore, maternal obesity and higher gestational weight gain possibly increase the risk of obesity and of non-communicable diseases in the offspring.^9^

Globally, adult height and BMI^10^^,^^11^ have been steadily increasing both for men and women, and obesity has reached epidemic proportions.^11^ In an earlier report comparing the Pelotas (Brazil) birth cohorts of 1982 and 2004, mean maternal height increased from 156.4 cm to 158.8 cm, and in all cohorts maternal height was positively associated with family income; the same period witnessed a marked increase in pre-pregnancy BMI. Maternal BMI was lower among the mothers in the extreme categories of family income, i.e. the poorest and wealthiest.^12^^,^^13^

In the present study, we report on: maternal height; pre-conceptional underweight, overweight and obesity; and gestational weight gain, according to socioeconomic position and maternal skin colour in the four population-based birth cohorts that were studied in the city of Pelotas (southern Brazil) in 1982, 1993, 2004 and 2015. We hypothesized that the trends observed from 1982 to 2004, particularly the increase in stature and in overweight and obesity,^12^ would continue to evolve in the period from 2004 to 2015. We focus on the description of time trends in levels and inequalities, rather than on the identification of other risk factors for anthropometric status.

## Methods

In 1982, 1993, 2004 and 2015, all maternity hospitals in Pelotas were visited daily and all children born to women who lived in the urban area of the city were examined, and their mothers were interviewed using a pre-coded questionnaire, soon after the delivery.^14^ Fewer than 1% of all births in the city took place outside a hospital, and subjects have been followed on several occasions; further details on the methodology of each birth cohort have been published elsewhere.^15–18^

In the four cohorts, information on maternal weight at the beginning of the pregnancy was obtained from the antenatal card, or through self-report if the information was not available on the card. Regarding maternal weight at the end of the pregnancy, in 1982 and 1993 women were weighed at hospital admission wearing light clothes and without shoes, using a scale (Filizola, precision 100 g) that was calibrated weekly by the research team using standard weights, and this information was abstracted by the research team from the maternity records. In 2004 and 2015, the mothers were asked about their weight at the end of the pregnancy during the perinatal interview. Concerning maternal height, in 1982 and 1993 mothers were measured at hospital admission by hospital staff and the research team retrieved this information from maternity records, whereas in 2004 and 2015 the mothers were measured at home, during the 3 months’ follow-up visit. In the four cohorts, all height measurements were carried out with the same model of a locally made portable stadiometer, with 1 mm precision.

Pre-pregnancy body mass index (in kg/m^2^) was calculated using the information on height and maternal weight at the beginning of the pregnancy. Overweight was defined by a body mass index at the beginning of the pregnancy ≥25 and <30 kg/m^2^, obesity by a BMI ≥30 kg/m^2^, overweight or obesity by a BMI >25 kg/m^2^and underweight by a BMI <18.5 kg/m^2^. Gestational weight gain was evaluated according to the Institute of Medicine (IOM) guidelines, which recommend weight gain ranges of 12.5–18.0 kg, 11.5–16.0 kg, 7–11.5  kg and 5.0–9.0 kg, among underweight, normal weight, overweight and obese mothers, respectively. For multiple pregnancies, we used the provisional IOM guidelines.^19^

The units of analyses were women who gave birth to a live-born child or to a stillbirth (a fetus with a gestational age of 28 or more weeks, or a birthweight of 1000 g or higher when gestational age was not known). Measurement procedures of birthweight and gestational age have been reported elsewhere.^20^ Single and multiple pregnancies were included.

Analyses were stratified by family income quintiles and maternal skin colour (white, brown or black). Further information on the stratification variables is available elsewhere.^14^ With respect to skin colour, in 1982 the interviewer classified maternal skin colour as white, black or other (either indigenous or yellow/Asian), and mothers with brown skin colour were classified as black. In 1993, the interviewer also classified the colour, and an option for brown skin color was included. In 2004 and 2015, skin colour was self-reported by the mothers using the five categories (white, brown, black, indigenous, yellow/Asian) employed by the Brazilian census bureau. Means and proportions were compared using analysis of variance (ANOVA) and chi square testing, respectively. Tests for heterogeneity and linear trends were carried out, and we reported the one with the lowest *P*-value of the two results. We compared differences inter- and intra-cohorts, i.e. in the inter-cohort comparisons we compared the change in the estimate across cohorts, for each category of the explanatory variable. For intra-cohort analysis, we estimated the likelihood that differences among the categories of the exposure variable were due to chance.

Two summary indices were used to assess the magnitude of income-related disparities.^21^ The slope index of inequality is a measure of absolute inequality and shows the difference in the outcome, expressed as percentage points, between the extremes of the wealth scale. The concentration index is a measure similar to the Gini coefficient, and reflects relative inequalities. Both indices range from −100 to +100, with positive values showing that the outcome is more common in the high-income group than in the poorest group. Both indices are based on the full distribution of the outcomes in the five wealth quintiles.^21^ A weighted least-square regression was used to carry out a formal statistical test of the variation in the concentration index and the slope index of inequality across the cohorts. Data analyses were carried out using Stata software 15.^22^

Ethical approval for observational studies was not required in Brazil until 1996.The 2004 study was approved by the Ethics Committee of the School of Medicine and the 2015 study by the Ethics Committee of the School of Physical Education, Federal University of Pelotas, and written informed consent was obtained from the mothers.

## Results

Response rates during the perinatal interview were greater than 98% in all four cohorts.^14^ From 1982 to 2015, the number of total births, including stillbirths, fell from 6011 to 4329. Additional information on the sociodemographic characteristics of the mothers in the four cohorts are presented elsewhere.^14^

Table 1 shows that average maternal height increased by 5.2 cm, from 156.4 cm in 1982 to 161.6 cm in 2015. The increment was slightly higher between 1982 and 1993 than from 2004 to 2015, whereas from 1993 to 2004 there was a slight decrease in height. Mean pre-pregnancy weight increased by 11.5 kg from 1982 to 2015, with the largest increment taking place between 2004 and 2015. Because the increase in weight was faster than that in height, a sharp increase in BMI was observed, mostly in the latest 11-year period. Whereas the prevalence of underweight declined from 7.8% to 3.7%, there was a marked increase in the prevalence of pre-pregnancy overweight or obesity, which affected about half of all women in 2015. Although mean gestational weight gain varied across the cohorts, there was no clear time trend and the means for 1982 and 2015 were similar. However, the proportion of mothers who gained more weight than recommended by the IOM guidelines was higher in 2004 and 2015, because a greater proportion of women were overweight or obese at the start of pregnancy—and therefore should have gained less weight.

**Table 1.** Maternal height, pre-pregnancy body mass index and weight gain during pregnancy in four birth cohorts, Pelotas, Brazil

	**Birth cohort (year)**^a^				*P*-value
	1982	1993	2004	2015	
Maternal height (cm)^b^	156.4	159.8	158.8	161.6	<0.01
	(156.3; 156.6)	(159.6; 160.0)	(158.5; 159.0)	(161.3; 161.8)	
	5902	5256	4011	4204	
Maternal height <150 cm (%)^c^	16.0	8.6	8.9	6.1	<0.01
	(15.1; 16.9)	(7.9; 9.4)	(8.1; 9.8)	(5.4; 6.9)	
Pre-pregnancy weight (kg)^b^	55.7	58.2	61.0	67.2	<0.01
	(55.5; 56.0)	(57.9; 58.5)	(60.6; 61.4)	(66.7; 67.6)	
	5146	5190	3998	4267	
Pre-pregnancy body mass	22.7	22.8	23.6	25.7	<0.01
index (kg/m^2^)^b^	(22.6; 22.8)	(22.7; 22.9)	(23.4; 23.7)	(25.5; 25.8)	
	5055	5147	3775	4152	
Pre-pregnancy underweight	7.8	8.8	7.4	3.7	<0.01
(BMI <18.5 kg/m^2^) (%)^c^	(7.0; 8.5)	(8.1; 9.6)	(6.5; 8.5)	(3.2; 4.3)	
Pre-pregnancy overweight (BMI	17.7	17.5	20.4	28.2	<0.01
≥25 and < 30 kg/m^2^) (%)^c^	(16.7; 18.8)	(16.5; 18.6)	(19.0; 21.9)	(26.9; 29.6)	
Pre-pregnancy obesity (BMI ≥	4.4	4.9	9.0	18.7	<0.01
30 kg/m^2^) (%)^c^	(3.9; 5.0)	(4.3; 5.5)	(8.0; 10.0)	(17.6; 20.0)	
Pre-pregnancy overweight or	22.1	22.4	29.4	47.0	<0.01
obesity (BMI ≥25 kg/m^2^) (%)^c^	(21.0; 23.3)	(21.2; 23.6)	(27.7; 31.0)	(45.5; 48.5)	
Gestational weight gain (kg)^b^	11.8	11.6	12.4	12.0	<0.01
	(11.7; 12.0)	(11.5; 11.8)	(12.2; 12.6)	(11.8; 12.2)	
	4468	5067	3949	4230	
Gestational weight gain according to IOM guidelines					<0.01
Below the recommendation (%)	41.0	42.6	27.5	30.8	
	(39.5; 42.4)	(41.2; 44.0)	(26.1; 28.9)	(29.4; 32.2)	
Within the recommendation (%)	34.4	33.8	32.4	33.5	
	(33.1; 35.9)	(32.5; 35.1)	(31.0; 33.9)	(32.1; 34.9)	
Above the recommendation (%)	24.6	23.6	40.1	35.7	
	(23.3; 25.9)	(22.5; 24.8)	(38.6; 41.6)	(34.3; 37.2)	

a 95% confidence interval is presented between brackets.

b Mean.

c Prevalence.

Table 2 shows mean maternal height in each cohort, disaggregated by quintiles of family income and maternal skin colour. In all four cohorts, maternal height was positively associated with family income. Mean height increased markedly over time in all socioeconomic groups, but the increase was greater in the two poorest quintiles (5.9 and 7.1 cm, respectively) than in the two richest quintiles (3.9 and 4.4 cm, respectively). The slope index of inequality and the concentration index suggest that the gap between the wealthiest and the poorest narrowed (*P* <0.01), mostly from 1982 to 1993. Regarding maternal skin colour, in 1993 and 2004 no differences in mean height were observed between white, brown and black women, and the increase in height over time was comparable in all three groups. On the other hand, in 1982 white mothers were taller than non-white mothers (*P* = 0.02), whereas in 2015 the 95% confidence intervals show that maternal height was higher among black and white mothers than for women with brown skin colour (*P* <0.01).

**Table 2.** Maternal height according to family income and skin colour in four birth cohorts, Pelotas, Brazil

	Mean maternal height in cm (95% confidence interval)				*P*-value
	1982	1993	2004	2015	
Quintiles of family income					
*P* -value	<0.01^a^	<0.01^b^	<0.01^b^	<0.01^b^	
Q1 (poorest)	154.1	158.1	157.5	160.0	<0.01^c^
	(153.8; 154.4)	(157.7; 158.5)	(157.0; 157.9)	(159.6; 160.5)	
Q2	153.7	159.2	157.7	160.8	<0.01^c^
	(153.4; 154.0)	(158.8; 159.6)	(157.3; 158.2)	(160.3; 161.2)	
Q3	157.2	159.5	158.5	161.2	<0.01^c^
	(157.0; 157.5)	(159.1; 160.0)	(158.1; 158.9)	(160.8; 161.6)	
Q4	158.0	160.6	159.0	161.9	<0.01^c^
	(157.7; 158.4)	(160.2; 161.0)	(158.6; 159.5)	(161.5; 162.3)	
Q5(wealthiest)	159.4	161.7	160.7	163.8	<0.01^c^
	(159.0; 159.7)	(161.3; 162.1)	(160.3; 161.1)	(163.4; 164.2)	
Concentration	0.75	0.40	0.38	0.44	<0.01
index	(0.69; 0.80)	(0.33; 0.46)	(0.31; 0.45)	(0.36; 0.51)	
Slope index of	1.60	0.76	0.76	0.76	<0.01
inequality	(1.49; 1.71)	(0.64; 0.88)	(0.63; 0.90)	(0.64; 0.88)	
Maternal skin colour				
*P*-value	0.0^a^	0.09^a^	0.30^a^	<0.01^a^	
White	156.5	159.8	158.7	161.9	<0.01^c^
	(156.4; 156.7)	(159.6; 160.0)	(158.5; 159.0)	(161.6; 162.1)	
Brown		158.9	158.2	159.9	<0.01^c^
	156.0^d^	(158.0; 159.8)	(157.5; 159.0)	(159.4; 160.5)	
Black	(155.7; 156.4)	160.0	158.5	161.5	<0.01^c^
		(159.6; 160.4)	(158.0; 158.9)	(160.9; 162.1)	

a *P*-value for heterogeneity from intra-cohort ANOVA tests.

b *P*-value for linear trend from intra-cohort ANOVA tests.

c *P*-value for heterogeneity from inter-cohort ANOVA tests.

d In 1982, brown women were classified as black; the results presented here expressed the mean height of black and brown women.

Maternal underweight prevalence is presented in Table 3. Except for 1982, there were inverse associations with family income. Important reductions over time were observed in all income groups, particularly among women in the richest quintiles. As a consequence, inequalities tended to increase slightly, particularly in relative terms as measured by the concentration index (*P* = 0.02). Regarding skin colour, we did not observe major differences across the cohorts.

**Table 3.** Prevalence of maternal underweight at the beginning of the pregnancy according to family income and skin colour in four birth cohorts, Pelotas, Brazil

	Prevalence of maternal underweight (95% confidence interval)				*P*-value
	1982	1993	2004	2015	
**Quintiles of family income**					
***P***-value	0.13^a^	<0.01^a^	<0.01^a^	<0.01^a^	
Q1 (poorest)	8.8	9.9	9.8	5.8	<0.01^b^
	(7.1; 10.9)	(8.2; 11.9)	(7.6; 12.7)	(4.4; 7.6)	
Q2	7.0	10.6	9.9	3.9	<0.01^b^
	(5.6; 8.8)	(8.9; 12.5)	(7.7; 12.8)	(2.8; 5.4)	
Q3	9.0	9.1	7.4	3.7	<0.01^b^
	(7.4; 10.9)	(7.4; 11.2)	(5.5; 9.9)	(2.6; 5.2)	
Q4	7.6	7.4	6.3	2.4	<0.01^c^
	(6.1; 9.3)	(5.9; 9.2)	(4.6; 8.5)	(1.5; 3.6)	
Q5(wealthiest)	6.5	6.4	4.6	3.0	<0.01^c^
	(5.1; 8.1)	(5.1; 8.1)	(3.2; 6.5)	(2.1; 4.4)	
Concentration	−4.53	−7.81	−15.25	−13.27	0.02
index	(−9.87; 0.80)	(−12.77; −2.85)	(−22.29; −8.21)	(−22.36; −4.18)	
Slope index of	−2.03	−4.27	−7.04	−3.40	0.41
inequality	(−4.55; 0.49)	(−6.96; −1.58)	(−10.38; −3.70)	(−5.54; −1.27)	
Maternal skin colour					
*P*-value	0.44^a^	0.20^a^	0.20^a^	0.63^a^	
White	7.9	8.9	7.4	3.6	<0.01^c^
	(7.1; 8.7)	(8.1; 9.8)	(6.4; 8.6)	(3.0; 4.3)	
Brown		11.6	10.2	4.4	<0.01^c^
	7.1^d^	(8.0; 16.4)	(6.8; 15.2)	(3.0; 6.5)	
Black	(5.5; 9.0)	7.8	6.4	3.9	<0.01^c^
		(6.3; 9.8)	(4.6; 8.8)	(2.6; 5.7)	

a *P*-value for heterogeneity from intra-cohort chi square tests.

b *P*-value for heterogeneity from inter-cohort chi square tests.

c *P*-value for linear trend from inter-cohort chi square tests.

d In 1982, brown women were classified as black; the results presented here expressed the prevalence of underweight among black and brown women.

In all four cohorts, the prevalence of pre-pregnancy overweight or obesity showed inverted U-shaped patterns according to income, with the highest prevalence in the intermediate quintiles (Table 4). Prevalence more than doubled between 1982 and 2015 in all but the second quintile. The slope and concentration indices do not show evidence of income-related inequality in maternal overweight or obesity in any cohort, as all confidence intervals included the reference (0). For maternal skin colour, the prevalence was lowest among white women in each cohort, but prevalence increased to similar extents in all groups over time. Table 5 shows that over the 33-year span, the prevalence of obesity increased by at least four times in all but the second quintile. Supplementary Figure 1 (available as Supplementary data at *IJE* online) shows that the prevalence of obesity presented an inverted U-shaped pattern with family income. We also analysed the trends for overweight (Supplementary Table 1, available as Supplementary data at *IJE* online).

**Table 4.** Prevalence of overweight or obesity at the beginning of the pregnancy according to family income and skin colour in four birth cohorts, Pelotas, Brazil

	Prevalence of overweight or obesity at the beginning of the pregnancy (95% confidence interval)				*P*-value
	1982	1993	2004	2015	
Quintiles of					
family income					
*P*-value	<0.01^a^	0.07^a^	<0.01^a^	<0.01^a^	
Q1 (poorest)	18.6	21.2	22.8	41.8	<0.01^c^
	(16.2; 21.3)	(18.7; 23.8)	(19.5; 26.6)	(38.4; 45.2)	
Q2	28.2	22.1	31.3	50.1	<0.01^b^
	(25.5; 31.1)	(19.9; 24.6)	(27.5; 35.3)	(46.6; 53.5)	
Q3	21.7	24.5	30.6	52.4	<0.01^c^
	(19.3; 24.2)	(21.8; 27.5)	(27.0; 34.6)	(49.0; 55.8)	
Q4	23.2	24.4	33.7	50.7	<0.01^c^
	(20.8; 25.8)	(21.8; 27.2)	(30.1; 37.5)	(47.3; 54.0)	
Q5(wealthiest)	18.7	20.0	27.8	39.8	<0.01^c^
	(16.4; 21.1)	(17.7; 22.6)	(24.5; 31.3)	(36.6; 43.2)	
Concentration	−2.52	0.02	3.12	−0.71	0.44
index	(−5.42; 0.39)	(−2.71; 3.08)	(0.00; 6.30)	(−2.56; 1.14)	
Slope index of	−3.00	0.11	5.08	−1.92	0.33
inequality	(−6.81; 0.88)	(−3.76; 3.98)	(−0.50; 10.65)	(−7.13; 3.29)	
Maternal skin colour					
*P*-value	<0.01^a^	0.03^a^	0.08^a^	<0.01^a^	
White	20.9	21.7	28.2	45.9	<0.01^c^
	(19.7; 22.1)	(20.4; 23.0)	(26.4; 30.2)	(44.2; 47.7)	
Brown		22.2	31.7	46.0	<0.01^c^
	28.5^d^	(17.3; 28.1)	(25.7; 38.4)	(41.8; 50.2)	
Black	(25.5; 31.6)	25.7	32.8	53.1	<0.01^c^
		(23.0; 28.6)	(29.0; 36.9)	(49.2; 57.0)	

a *P*-value for heterogeneity from intra-cohort chi square tests.

b *P*-value for heterogeneity from inter-cohort chi square tests.

c *P*-value for linear trend from inter-cohort chi square tests.

d In 1982, brown women were classified as black; the results presented here expressed the prevalence of overweight or obesity among black and brown women.

**Table 5.** Prevalence of obesity at the beginning of the pregnancy according to family income and skin colour in four birth cohorts, Pelotas, Brazil

	Prevalence of obesity at the beginning of the pregnancy (95% confidence interval)				*P*-value
	1982	1993	2004	2015	
Quintiles of family income					
*P*-value	<0.01^a^	0.10^a^	0.51^a^	<0.01^a^	
Q1 (poorest)	3.6	4.0	8.1	17.8	<0.01^c^
	(2.5; 5.0)	(3.0; 5.5)	(6.1; 10.8)	(15.3; 20.5)	
Q2	6.7	5.1	10.1	20.3	<0.01^b^
	(5.3; 8.4)	(3.9; 6.5)	(7.9; 13.0)	(17.7; 23.2)	
Q3	4.8	5.7	9.9	21.3	<0.01^c^
	(3.7; 6.3)	(4.4; 7.5)	(7.7; 12.6)	(18.6; 24.1)	
Q4	4.5	5.9	9.1	20.8	<0.01^c^
	(3.4; 5.9)	(4.6; 7.5)	(7.1; 11.7)	(18.2; 23.7)	
Q5(wealthiest)	2.5	3.7	7.7	13.6	<0.01^c^
	(1.7; 3.7)	(2.7; 5.1)	(5.9; 10.0)	(11.4; 16.0)	
Concentration	−8.6	0.12	−1.31	−3.70	0.55
index	(−15.20; −2.02)	(−6.49; 6.73)	(−7.83; 5.22)	(−7.20; −0.21)	
Slope index of	−2.37	−0.02	−1.19	−3.93	0.96
inequality	(−4.18; -0.58)	(−1.92; 1.88)	(−4.66; 2.27)	(−7.84; −0.01)	
Maternal skin					
colour					
*P*-value	<0.01^a^	0.14^a^	0.01^a^	0.22^a^	
White	3.9	4.5	8.1	18.2	<0.01^c^
	(3.4; 4.6)	(3.9; 5.2)	(7.0; 9.3)	(16.8; 19.6)	
Brown		5.8	13.2	19.3	<0.01^c^
	7.0^d^	(3.3; 9.7)	(9.2; 18.5)	(16.2; 22.8)	
Black	(5.4; 8.9)	6.0	10.8	21.1	<0.01^c^
		(4.7; 7.7)	(8.4; 13.7)	(18.1; 24.5)	

a *P*-value for heterogeneity from intra-cohort chi square tests.

b *P*-value for heterogeneity from inter-cohort chi square tests.

c *P*-value for linear trend from inter-cohort chi square tests.

d In 1982, brown women were classified as black; the results presented here expressed the prevalence of obesity among black and brown women.

In all cohorts except for 2015, the proportion of mothers whose weight gain was above the recommended range (Table 6) was higher in the top income quintile, but the fastest increase was observed in the poorest quintiles. Accordingly, the concentration and slope indices show important declines in inequalities over time (*P* < 0.01). From 1982 to 2004 weight gain was not associated with skin colour, but in 2015 the proportion of mothers with a weight gain above the recommendation was lower among white women compared with other women.

**Table 6.** Prevalence of weight gain during pregnancy above the recommended range, according to family income and skin colour in four Birth Cohorts. Pelotas, Brazil

	Percentage weight gain during pregnancy above the recommendations (95% confidence interval)				*P*-value
	1982	1993	2004	2015	
Quintiles of					
family income					
*P*-value	<0.01^b^	<0.01^a^	<0.01^b^	<0.01^a^	
Q1 (poorest)	15.7	21.9	33.5	30.1	<0.01^c^
	(13.2; 18.6)	(19.4; 24.6)	(30.2; 37.0)	(27.1; 33.4)	
Q2	21.1	20.8	38.9	39.4	<0.01^c^
	(18.5; 24.0)	(18.5; 23.2)	(35.5; 42.3)	(36.2; 42.8)	
Q3	24.4	22.8	40.9	36.5	<0.01^c^
	(21.8; 27.3)	(20.1; 25.7)	(37.5; 44.4)	(33.4; 39.8)	
Q4	25.6	24.6	43.3	36.1	<0.01^c^
	(22.9; 28.4)	(22.1; 27.4)	(39.9; 46.7)	(32.9; 39.4)	
Q5(wealthiest)	33.3	28.6	43.4	36.3	<0.01^c^
	(30.4; 36.3)	(25.9; 31.4)	(40.0; 46.8)	(33.2; 39.6)	
Concentration	13.22	5.70	4.72	1.82	<0.01
index	(10.31; 16.12)	(2.83; 8.57)	(2.53; 6.91)	(−0.50; 4.13)	
Slope index of	18.92	7.75	11.60	4.24	<0.01
inequality	(14.63; 23.21)	(3.68; 11.83)	(6.38; 16.83)	(−0.71; 9.18)	
Maternal skin colour					
*P*-value	0.51^a^	0.18^a^	0.26^b^	0.04^b^	
White	24.8	24.1	40.6	34.8	<0.01^c^
	(23.4; 26.2)	(22.8; 25.5)	(38.8; 42.4)	(33.1; 36.5)	
Brown		19.4	39.2	37.8	<0.01^c^
	23.6^e^	(14.7; 25.3)	(33.6; 45.1)	(33.8; 41.9)	
Black	(20.6; 26.9)	22.4	38.4	38.6	<0.01^d^
		(19.8; 25.2)	(35.0; 41.9)	(34.9; 42.4)	

a *P*-value for heterogeneity from intra-cohort chi square tests.

b *P*-value for linear trend from intra-cohort chi square tests.

c *P*-value for heterogeneity from inter-cohort chi square tests.

d *P*-value for linear trend from inter-cohort chi square tests.

e In 1982, brown women were classified as black; the results presented here expressed the mean weight gain during pregnancy of black and brown women.

## Discussion

The strengths of our analyses include the population-based nature of the samples. Each perinatal study included nearly all births in a calendar year and response rates were above 98%, thus minimizing the likelihood of selection bias. The four studies were conducted by the same group of researchers. The study’s limitations included differences in the assessment of maternal weight. Whereas in 1982 and 1993 women were weighed at hospital admission, in 2004 and 2015 the information on the weight at the end of the pregnancy was provided by the mothers, who usually reported their weight at the latest antenatal care visit. This change in the way of assessing maternal weight may have biased our analysis of the trend of gestational weight gain. Moreira and colleagues studied the agreement between self-reported and measured weights in the 2013 national health survey in Brazil, showing that there was a high degree of agreement between both variables.^23^ Headen *et al.*^24^ systematically reviewed the evidence on the accuracy of self-reported maternal weight across pregnancy. Concerning weight at the end of pregnancy, most studies reported errors of small magnitude, with mothers tending to under-report their weight. Because the last antenatal care visit usually takes place before the day of delivery, in 2004 and 2015 we did not capture the change in weight between the antenatal care visit and the delivery, and thus weight gain during pregnancy was underestimated. Since this error is expected to be independent of socioeconomic status, non-differential misclassification bias may be present. This will tend to underestimate the associations with risk factors in 2004 and 2015. Other limitations, regarding the collection of information on family income and skin colour, are further discussed in the first article in this supplement.^14^ The misclassification of family income that may have occurred in 1993, due to the hyperinflation, tends to be unrelated to maternal nutritional status and this error may have underestimated the association of nutritional status with socioeconomic status in 1993. By the same token, the change in the assessment of skin colour (as the ‘brown’ category was not considered in 1982) may have introduced some noise in the observed associations.

In the 33-year period covered by the four birth cohorts, there were important changes in sociodemographic characteristics of the mothers, which are described in an accompanying article.^14^ There were important improvements in education and income. The proportion of adolescent mothers remained stable, but there was a substantial increase in the number of mothers aged 35 years or older. Parity declined rapidly, and birth intervals increased.

The present results show that average maternal height and pre-pregnancy weight increased markedly. Because the increase was faster for weight than for height, the prevalence of pre-pregnancy overweight or obesity rose from 22.1% in 1982 to 47.0% in 2015. The fastest increase took place after 2004. On the other hand, weight gain during pregnancy did not change across the cohorts, but this could be at least partly due to the above-mentioned differences in how the final weight was assessed in 2004 and 2015.

Both small stature and underweight were more common among low-income women. Inequalities in maternal heights according to income fell rapidly particularly between 1982 to 1993 but, in contrast, inequalities in underweight increased slightly due to faster reductions in the richest quintiles where the prevalence is rather low. In contrast, overweight or obesity did not show a linear association with income, and inequalities in overweight or obesity were small and remained stable during the study period, as prevalence increased in all quintiles. Weight gains during pregnancy above the recommendations were more frequent among high-income women in 1982, but by 2015 these differences were markedly reduced due to faster increase among the poor. Generally speaking, women from all income groups were more similar in terms of anthropometric status in 2015 than they were in 1982. On the one hand this is a positive finding, as is the case for height, but on the other hand improved equality was due to the faster rises among the poor in overweight or obesity and weight gain during pregnancy above the recommendation. When equity improves as a function of worsening status among the poor, the improvement is illusory.

The findings on maternal anthropometry are consistent with the reduction in stunting and the increase in overweight or obesity in the children from the four cohorts, which are described in another article in this supplement.^25^ Changes in inequalities were also similar for mothers and children: the socioeconomic gap in stunting was greatly reduced, but the faster increase in overweight or obesity among poor children led to the elimination of the gap that was present in 1982.

The findings from our four cohorts are consistent with the global increase in overweight and obesity, which has reached epidemic levels in several countries.^11^ Increases in the prevalence of overweight and obesity in the beginning of pregnancy have been described in high-income countries,^26–28^ where several studies report higher prevalence of obesity among mothers of low socioeconomic status.^28^^,^^29^ In contrast, studies from low-income countries show higher prevalence among wealthy women, although this pattern is changing rapidly.^30^ A study of national trends in Brazil up to 2008^31^ showed that the prevalence of obesity was increasing faster among the poor than among the rich women. In contrast, we found that although the absolute increase in percentage points was greater for poor than for rich women (Supplementary Figure 1), there were similar 5-fold increases in prevalence in both the poorest and richest quintiles. Both the national study and our own findings show that the prevalence of obesity was highest among women in the intermediate categories of socioeconomic status. This pattern of association did not change over time. On the other hand, similarly to other settings, black mothers were more likely to be overweight.^28^^,^^29^

As observed in other countries, we documented an important increase in maternal height over time. This increase in height in our birth cohorts was more pronounced in two different periods, from 1982 to 1993 and from 2004 to 2015. Over the 33-year span, mean maternal height increased by about 5 cm and the proportion of mothers whose height was <150 cm decreased from 16.0% in 1982 to 6.0% in 2015. Though an increase in adult height has been reported worldwide,^10^ an analysis of data from 54 low- to middle-income countries showed that in 35 of them a stagnation or decline in female height has been documented in most recent birth cohorts,^32^ with the increase in height being concentrated among women in the wealthiest socioeconomic groups in more recent years.

In Pelotas, the slope index of inequality and the concentration index make clear that the difference in maternal height between the richest and the poorest narrowed, mostly from 1982 to 1993, which indicates that socioeconomic inequalities in terms of maternal height decreased. The improvements in maternal height which we documented in our cohorts have also been observed for height of young children; Gonçalves *et al*. reported a marked decrease in the prevalence of stunting at 12 months of age,^25^ as well as reductions in socioeconomic inequalities. In Brazil, the prevalence of stunting in childhood decreased from 37.1% in 1974–75 to 7.1% in 2006–7.^33^ Because undernutrition in childhood is positively associated with stature in adulthood,^3^ such improvement in maternal height was expected and should be associated with further improvements in the next generation, as early growth is associated with intrauterine growth in the next generation.^34^

This analysis showed positive trends in maternal height and socioeconomic inequality which increased and declined, respectively. On the other hand, the increase in maternal overweight or obesity is a cause for concern, given its short- and long-term consequences on the health of the mother and the baby. These findings reinforce the need for ample public health policies aimed at tackling the obesity epidemic.

## Funding

The four cohorts received funding from the following agencies: Wellcome Trust, International Development Research Center, World Health Organization, Overseas Development Administration of the United Kingdom, European Union, Brazilian National Support Program for Centers of Excellence (PRONEX), Brazilian National Council for Scientific and Tehcnological Development (CNPq), Science and Technology Department (DECIT) of the Brazilian Ministry of Health, Research Support Foundation of the State of Rio Grande do Sul (FAPERGS), Brazilian Pastorate of the Child and Brazilian Association for Collective Health (ABRASCO).

## Pelotas Cohorts Study Group

Aluisio J D Barros,^1^ Alicia Matijasevich,^2^ Ana M B Menezes,^1^ Andrea Dâmaso Bertoldi,^1^ Diego G Bassani,^3^ Fernando C Wehrmeister,^1^ Helen Gonçalves,^1^ Joseph Murray,^1^ Luciana Tovo-Rodrigues,^1^ Mariangela F Silveira^1^ and Pedro R C Hallal.^1^

^1^Federal University of Pelotas, Brazil, ^2^University of São Paulo, Brazil and ^3^University of Toronto, Canada.

**Conflict of interest:** None declared.

## Supplementary Material

Supplementary Data
