# Supplementary material for: Maternal anthropometry: trends and inequalities in four population-based birth cohorts in Pelotas, Brazil, 1982–2015
Source: Int J Epidemiol. 2019 Mar 18;48(Suppl 1):i26–36. doi: 10.1093/ije/dyy278 (PMC6422063; doi:10.1093/ije/dyy278)
Supplement: Supplementary Data [file dyy278_supp.zip › dyy278_Suppl_data/dyy278_Supplementary_Table.docx]

Supplementary Table. Prevalence of overweight at the beginning of the pregnancy according to family income and skin color in four Birth Cohorts. Pelotas, Brazil.

|  | Prevalence of overweight at the beginning of the | | | | *p-value* |
| --- | --- | --- | --- | --- | --- |
|  | pregnancy (95% confidence interval) | | | |  |
|  | 1982 | 1993 | 2004 | 2015 |  |
| Quintiles of |  |  |  |  |  |
| family income |  |  |  |  |  |
| *p-value* | *< 0.01 ^a^* | *0.55 ^a^* | *< 0.01 ^a^* | *< 0.01 ^a^* |  |
| Q1 (poorest) | 15.0 | 17.1 | 14.7 | 24.0 | *< 0.01 ^b^* |
|  | (12.8; 17.5) | (14.9; 19.6) | (11.9; 18.0) | (21.2; 27.1) |  |
| Q2 | 21.5 | 17.1 | 21.2 | 29.8 | *< 0.01 ^b^* |
|  | (19.1; 24.2) | (15.0; 19.4) | (17.9; 24.8) | (26.7; 33.0) |  |
| Q3 | 16.8 | 18.8 | 20.8 | 31.2 | *< 0.01 ^c^* |
|  | (14.7; 19.2) | (16.4; 21.6) | (17.6; 24.3) | (28.1; 34.4) |  |
| Q4 | 18.7 | 18.5 | 24.6 | 29.8 | *< 0.01 ^c^* |
|  | (16.5; 21.1) | (16.4; 21.6) | (21.3; 28.1)) | (26.8; 33.0) |  |
| Q5(wealthiest) | 16.2 | 16.3 | 20.1 | 26.3 | *< 0.01 ^c^* |
|  | (14.1; 18.6) | (14.1; 18.7) | (17.2; 23.3) | (23.4; 29.3) |  |
| Concentration | -0.69 | 0.79 | 4.79 | 1.06 | *0.34* |
| index | (-4.04; 2.65) | (-2.59; 4.16) | (0.82; 8.76) | (-1.67; 3.78) |  |
| Slope index of | -0.59 | 0.13 | 6.27 | 2.01 | *0.13* |
| inequality | (-4.16; 2.96). | (-3.43; 3.69) | (1.38; 11.16) | (-2.60; 6.62) |  |
| Maternal skin |  |  |  |  |  |
| color |  |  |  |  |  |
| *p-value* | *< 0.01 ^a^* | *0.17  ^a^* | *0.48 ^a^* | *0.07 ^a^* |  |
| White | 16.9 | 17.1 | 20.2 | 27.7 | < 0.01 *^c^* |
|  | (5.8; 18.1) | (16.0; 18.3) | (18.5; 21.9) | (26.2; 29.4) |  |
| Brown |  | 16.4 | 18.5 | 26.7 | < 0.01 *^c^* |
|  | 21.5 ^d^ | (12.2; 21.9) | (13.8; 24.5) | (23.1; 30.5) |  |
| Black | (18.8; 24.4) | 19.7 | 22.1 | 32.0 | < 0.01 *^c^* |
|  |  | (17.2; 22.3) | (18.8; 25.7) | (28.5; 35.8) |  |

^a^ p-value for heterogeneity from intra-cohort chi-squared tests

^b^ p-value for heterogeneity from inter-cohorts chi-squared tests

^c^ p-value for linear trend from inter-cohorts chi-squared tests

^d^ In 1982, brown women were classified as black, the results presented here expressed the prevalence of overweight at the beginning pregnancy of black and brown women.
