# Supplementary material for: Maternal anthropometry: trends and inequalities in four population-based birth cohorts in Pelotas, Brazil, 1982–2015
Source: Int J Epidemiol. 2019 Mar 18;48(Suppl 1):i26–36. doi: 10.1093/ije/dyy278 (PMC6422063; doi:10.1093/ije/dyy278)
Supplement: Supplementary Data [file dyy278_supp.zip › dyy278_Suppl_data/dyy278_Supplementary_Figure.docx]

Supplementary Figure 1. Prevalence of obesity at the beginning of the pregnancy according to family income in four Birth Cohorts. Pelotas, Brazil.

Prevalence of obesity (%)
